# Breaking the serologic rule: chronic hepatitis B with simultaneous HBsAg and anti-HBs positivity—a case report

**DOI:** 10.1186/s13256-026-06091-y

**Published:** 2026-05-07

**Authors:** Ghada Mohamed Balah, Mohammed Salmen Bazuqamah, Eslam Ahmed Helal, Mohammad Aly Moharram, Hend Masoud Masoud, Amira Abdelfatah Soliman, Ali Ismail Yahya

**Affiliations:** 1 Immunology & Virology Laboratory Department, King Khalid Hospital, King Abdulaziz Road, 66261 Najran, Saudi Arabia; 2 Microbiology Laboratory Department, King Khalid Hospital, Najran, Saudi Arabia; 3 Biochemistry Laboratory Department, King Khalid Hospital, Najran, Saudi Arabia; 4 Hematology Laboratory Department, King Khalid Hospital, Najran, Saudi Arabia; 5 Blood Bank Laboratory Department, King Khalid Hospital, Najran, Saudi Arabia

**Keywords:** Chronic hepatitis B, HBsAg, Anti-HBs, Concurrent HBsAg and anti-HBs, Diagnostic pitfall, Hepatitis B virus DNA, Case report

## Abstract

**Background:**

Chronic hepatitis B virus (HBV) infection continues to pose a significant global disease burden, because of its potential progression to liver cirrhosis and hepatocellular carcinoma (HCC). Worldwide, more than 250 million individuals are estimated to be living with chronic HBV infection**.** Under standard serologic interpretation, the presence of hepatitis B surface antigen (HBsAg) signifies ongoing infection, whereas antibodies to hepatitis B surface antigen (anti-HBs) are generally associated with viral clearance or protective immunity. The simultaneous presence of both markers is rare and may lead to misinterpretation of serologic results, particularly when identified incidentally during routine screening in a primary care settings.

**Case presentation:**

We describe a 60-year-old Saudi woman with an asymptomatic chronic HBV infection incidentally identified during routine pre-colonoscopy screening in a primary care setting. Laboratory testing revealed reactive HBsAg, total anti-HBc positivity, reactive anti-HBs titer, and HBV DNA level was 275 IU/mL consistent with low-level viremia. Liver enzymes were mildly elevated on prior testing subsequently normalized on follow- up. Transient elastography demonstrated no evidence of hepatic fibrosis or steatosis. The patient had no history of HBV vaccination, blood transfusions, liver disease, or recognized high-risk exposures, including intravenous drug use or high-risk sexual or household contact.

**Conclusion:**

This case highlights a diagnostic pitfall in HBV serology, as concurrent HBsAg and anti-HBs positivity does not indicate viral clearance and may reflect immune escape mutations, viral heterogeneity, or host immune factors. Recognition of this rare serologic profile is particularly important for primary care physicians, as accurate interpretation requires integration of serologic, virologic, and clinical data, particularly in asymptomatic patients identified through routine screening. This ensure appropriate diagnosis, long-term surveillance, and risk stratification. Clinicians should remain vigilant when encountering this rare serologic profile to guide appropriate referral and follow-up.

## Background

Globally, chronic HBV infection affects hundreds of millions of individuals and remains a leading cause of liver-related morbidity and mortality, that represents a substantial public health challenge, with outcomes spanning from acute self-limiting infection to chronic hepatitis, liver cirrhosis, and hepatocellular carcinoma (HCC). These outcomes are largely determined by the interplay between viral factors and the host immune response [1]. Typically, active HBV infection is indicated by the presence of hepatitis B surface antigen (HBsAg) in the blood, whereas the development of neutralizing antibodies against HBsAg (anti-HBs) generally signifies resolution of infection, either spontaneously or following antiviral therapy, or immunity acquired through vaccination [2].

In rare instances, HBsAg and anti-HBs coexist simultaneously, creating a paradoxical serologic profile that poses diagnostic and clinical challenges, particularly when identified incidentally during routine screening in asymptomatic individuals [3, 4]. This unusual pattern may result from immune escape variants, viral mutations, infection with heterologous HBV subtypes, or altered host immune responses [3, 5]. Among patients with chronic hepatitis B (CHB), the coexistence of HBsAg and anti-HBs has been associated with an increased risk of HCC and may influence disease progression and long-term prognosis. This pattern is associated with a higher incidence of HCC, with one study showing a 5-year cumulative incidence of 12.7% in the coexistence group versus 4.9% in those with HBsAg alone [6, 7].

We report the case of a 60-year-old anasymptomatic woman with chronic HBV infection incidentally identified during routine pre-procedural screening, demonstrating concurrent positivity for HBsAg and anti-HBs, to highlight this diagnostic challenge and discuss its potential mechanisms and clinical implications of this rare serologic pattern for both frontline and specialist clinicians.

## Case presentation

A 60-year-old Saudi woman presented to the outpatient department at King Khalid Hospital, Najran, with intermittent, nonspecific abdominal pain. She had no symptoms suggestive of acute hepatitis or liver disease. She denied HBV vaccination, blood transfusions, intravenous drug use, high-risk sexual behavior, alcohol consumption, or immunosuppressive therapy, confirming no identifiable high-risk exposures. During a routine primary care visit for colorectal cancer screening, a fecal immunochemical test (FIT) was positive, prompting referral for colonoscopy. Colonoscopic evaluation revealed no significant abnormalities, and histopathologic examination demonstrated no evidence of dysplasia or malignancy.

Routine pre-procedural laboratory investigations revealed the following results: ALT 22 U/L (normal 1–34), AST 22 U/L (normal 1–34), alkaline phosphatase 143 U/L (normal 30–120), GGT 15 U/L (normal 1–37), total bilirubin 59.2 µmol/L (normal 5–21), direct bilirubin 9.4 µmol/L (normal 0–3.39), and albumin 37 g/L (normal 35–52). Three months prior, mild elevations were noted: ALT 56 U/L, AST 60 U/L, ALP 127 U/L, GGT 12 U/L, total bilirubin 49.4 µmol/L, direct bilirubin 10.1 µmol/L, and albumin 33 g/L (Table 1).

**Table 1:** Laboratory findings of the patient

Parameters	3 months before colonoscopy	Pre-colonoscopy	4 months follow up	Reference range
ALT	**56** **U/L**	22 U/L	17 U/L	1–34 U/L
AST	**60** **U/L**	22 U/L	22 U/L	1–34 U/L
ALP	**127** **U/L**	**143** **U/L**	**142** **U/L**	30–120 U/L
GGT	12 U/L	15 U/L	18 U/L	1–37 U/L
Total bilirubin	**49.4 µmol/L**	**59.2 µmol/L**	**55.1 µmol/L**	5–21 µmol/L
Direct bilirubin	**10.1 µmol/L**	**9.4 µmol/L**	**8.8 µmol/L**	0–3.39 µmol/L
Albumin	**33 g/L**	37 g/L	42 g/L	35–52 g/L

*AST* Aspartate Aminotransferase, *ALT* alanine aminotransferase, *GGT* gamma-glutamyl transferase, *ALP* Alkaline phosphatase

Bold text indicates abnormal values

Serologic testing using a chemiluminescence immunoassay showed reactive HBsAg, reactive anti-HBs titer (11.2 mIU/mL; reference range: reactive ≥ 10 mIU/mL), total anti-HBc positivity, and non-reactive anti-HBc IgM. Hepatitis C virus antibodies were negative. Quantitative HBV DNA polymerase chain reaction (PCR) demonstrated low-level viremia at 275 IU/mL. The assay detection limit was 20 IU/mL, confirming that the observed level reflects low but detectable viral replication (Table 2).

**Table 2:** Key diagnostic tests (pre-colonoscopy)

Test	Result	Reference/Interpretation
HBsAg	Reactive	Indicates ongoing infection
Anti-HBs	11.2 mIU/mL	Low-titer positivity (≥ 10 mIU/mL reactive)
Total anti-HBc	Reactive	Confirms prior exposure
Anti-HBc IgM	Non-reactive	No acute infection
HBV DNA	275 IU/mL	Low-level viremia (detection limit 20 IU/mL)
FibroScan	3.0 kPa/CAP 100 dB/m	No fibrosis or steatosis

Abdominal ultrasonography revealed a post-cholecystectomy status with otherwise normal hepatic and biliary anatomy, no focal lesions, and no evidence of ascites or lymphadenopathy.

Transient elastography (FibroScan^®^) demonstrated a liver stiffness measurement of 3.0 kPa (F0, indicating no fibrosis) and a controlled attenuation parameter (CAP) of 100 dB/m (S0, indicating no steatosis), with an interquartile range (IQR) of 30%, consistent with acceptable scan reliability (Table 2).

Based on serologic and virologic findings, a diagnosis of chronic hepatitis B infection with the atypical coexistence of HBsAg and anti-HBs was established. Antiviral therapy was deferred due to low-level viremia and normal liver function, she was referred to hepatology for further evaluation, including consideration of HBV genotyping and long-term monitoring.

A follow-up plan was established: routine monitoring in hepatology clinic every 6–12 months, including assessment of liver enzymes, HBV DNA, and HBsAg/anti-HBs levels, as well as surveillance for fibrosis progression and hepatocellular carcinoma.

This case highlights the incidental detection of chronic hepatitis B in a primary care setting, illustrating how routine pre-procedural screening can uncover atypical serologic patterns. It underscores the importance of clinician awareness regarding low-level viremia and the need for careful interpretation of HBV serology, particularly in asymptomatic patients without known risk factors.

**Table:** Timeline

Time point	Clinical event
3 months prior	Mild elevation of ALT, AST, and bilirubin detected on routine laboratory testing
Screening visit	Positive fecal immunochemical test (FIT)
Pre-colonoscopy evaluation	HBsAg positive, anti-HBs positive (11.2 mIU/mL), HBV DNA 275 IU/mL
Imaging	Abdominal ultrasound normal; FibroScan showed F0 fibrosis and S0 steatosis
Current status	Normal liver enzymes; referred to hepatology for monitoring
4 months follow up	Normal ALT/AST, AFP within normal range; abdominal ultrasound showed no focal hepatic lesions or ascites

### Follow-up and outcomes

The patient remains clinically stable under regular hepatology follow-up, with normal liver enzyme levels and there is no clinical evidence of hepatic decompensation or disease progression.

At 4 months of follow-up, repeat laboratory evaluation showed normal transaminases (ALT 17 U/L, AST 21 U/L), with mildly elevated total bilirubin (55.1 µmol/L) and alkaline phosphatase (142 U/L) [Table 1]. Alpha-fetoprotein was within normal limits 2.2 ng/mL (normal: 0–9). Follow-up abdominal ultrasonography demonstrated a liver of normal size and echotexture, without focal lesions, biliary dilatation, ascites, or abnormal portal vein caliber.

These findings indicate ongoing biochemical and radiologic stability. The patient continues routine surveillance for chronic hepatitis B and hepatocellular carcinoma (HCC),

## Discussion

The coexistence of HBsAg and anti-HBs represents a paradoxical serologic pattern that challenges the conventional interpretation of hepatitis B virus (HBV) infection [6, 8]. Although traditionally considered mutually uncommon, this phenomenon is increasingly recognized in clinical practice and may be underappreciated during routine screening, particularly in a primary care setting. Conventionally, the presence of HBsAg denotes active viral replication, although anti-HBs is considered a marker of recovery or vaccine-induced immunity [9, 10]. Their simultaneous presence suggests complex interactions between viral characteristics and host immune responses and carries important diagnostic and clinical implications [3].

The prevalence of concurrent HBsAg and anti-HBs positivity may reach up to 4.2% in certain populations, with considerable geographic variation. Reported prevalence ranges from 2.4 to 5.8% in China, 2.9–7.0% in South Korea, 5.0–8.9% in France, and 0.2–3.6% in Turkey [11, 12]. Previous studies have further shown that individuals with concurrent anti-HBs positivity tend to be older, have lower platelet counts, and demonstrate a higher prevalence of hepatitis B e antigen (HBeAg) positivity. In addition, this serologic pattern has been associated with significantly lower circulating HBsAg levels, indicating a potentially distinct virological or immunological phenotype [13]. These findings suggest that this serologic pattern is uncommon overall but may be more frequently observed in regions with high HBV endemicity, likely reflecting differences in HBV genotypes, host immunity, vaccination coverage, and diagnostic methodologies. Data on racial and ethnic predisposition remain limited, but available studies indicate higher reporting rates in Asian and Mediterranean populations, consistent with regional HBV epidemiology [9], from a clinical and public health perspective, these findings are important. They underscore the continued importance of hepatitis B vaccination programs, especially in regions with high endemicity, as vaccination remains the most effective strategy for preventing HBV transmission and reducing the overall burden of disease. Careful assessment of vaccination history and comprehensive serologic testing are therefore essential to ensure accurate diagnosis and optimal patient management.

Several pathogenic and immunologic mechanisms have been proposed to account for this serologic phenomenon. One of the most widely accepted explanations involves *Immune Escape Mutations* within the “a” determinant region of the HBV surface (S) gene—most notably the G145R mutation—can alter the antigenic structure of HBsAg, reducing recognition by anti-HBs antibodies [4, 5]. These immune escape variants have been well documented in patients with chronic HBV infection, including those with vaccine breakthrough infections or prior antiviral therapy. In previously reported cases, genotypic analysis has frequently identified such mutations; however, HBV genotyping was not performed in our patient, representing a notable limitation of this case.

Another proposed mechanism relates to viral heterogeneity and host immune factors. Superinfection with a heterologous HBV subtype may result in the production of anti-HBs antibodies directed against one serotype, although a different circulating HBsAg subtype persists unchecked. In this scenario, the generated antibodies fail to effectively neutralize non-homologous HBsAg, allowing continued viral replication despite apparent serologic immunity [14].

Age-related viral evolution may also contribute to this phenomenon. Several studies have demonstrated that amino acid substitutions within the HBV genome accumulate over time and are more frequently observed in older individuals [9, 10]. This progressive viral variability may facilitate immune escape and increase the likelihood of concurrent HBsAg and anti-HBs positivity, which may partially explain the observed serologic pattern in our 60-year-old patient.

In the present case, the patient demonstrated reactive HBsAg, total anti-HBc, and anti-HBs positivity, with low level but detectable HBV DNA levels, confirming chronic HBV infection despite the presence of anti-HBs. Previous studies by Wang et al. [6], Zhang et al. [15] have shown that the anti-HBs positivity is often associated with lower HBsAg levels, this is consistent with our findings. Furthermore, Jin et al. [7] reported that patients with low-level HBsAg and concurrent anti-HBs carry an increased risk of HCC when compared with those lacking anti-HBs, underscoring the clinical relevance of quantifying these markers. Although long-term outcome data for our patient are limited, these findings underscore the risk of diagnostic misinterpretation and false reassurance if HBV serologic markers are interpreted in isolation.

This case adds to the existing literature by demonstrating how an atypical serologic pattern can be incidentally identified during routine pre-procedural screening in a primary care settings, where it carries a substantial risk of misinterpretation and delayed diagnosis. The finding highlights a common clinical scenario in primary care and underscores the importance of routine screening even in asymptomatic individuals. Notably, the patient had no history of recognized high-risk exposures, including blood transfusion, intravenous drug use, or household contact with hepatitis B virus, emphasizing that chronic infection may remain clinically silent and undetected in the absence of systematic testing.

From a clinical standpoint, low-level viremia, as documented by HBV DNA above the assay detection limit (20 IU/mL), warrants regular monitoring, including liver function tests and imaging for early detection of disease progression. Short-term follow-up at 4 months demonstrated biochemical and radiologic stability with normal transaminases, normal alpha-fetoprotein levels, and an unremarkable abdominal ultrasound. Although reassuring, this does not exclude long-term risk, and continued surveillance remains essential. In cases with atypical serologic patterns, HBV genotyping may provide additional prognostic information, particularly regarding potential immune escape mutations and long-term hepatocellular carcinoma risk.

Taken together, this case highlights an important clinical principle: the presence of anti-HBs alone should not be assumed to indicate viral eradication or vaccination. Accurate serologic interpretation requires integration of virologic data, particularly in the presence of detectable HBsAg or HBV DNA. Comprehensive evaluation, including HBV DNA quantification and fibrosis assessment, is essential to accurately characterize disease status, prevent false reassurance, and guide appropriate clinical management. For primary care physicians, awareness of this rare serologic profile and the importance of routine pre-procedural HBV screening, can uncover unexpected chronic infections even in asymptomatic patient without known risk factors which helps ensure timely referral and appropriate longitudinal follow-up.

### Study limitations

This case has several limitations. First, HBV genotyping and mutation analysis were not performed, limiting our ability to confirm immune escape mutations. Second, the evaluation was based on a single time point, with no longitudinal follow-up. Serial monitoring of HBV DNA, HBsAg, and anti-HBs levels would be valuable to assess disease evolution, serologic changes, and long-term outcomes. Future studies could address these gaps.

### Clinical implications

Clinicians should be aware of the diagnostic pitfall posed by concurrent HBsAg and anti-HBs positivity, as this serologic pattern does not exclude chronic hepatitis B infection. Evaluation should include HBV DNA quantification and assessment of hepatic fibrosis, with referral to a specialist for appropriate risk stratification and longitudinal monitoring. In patients with atypical serologic profiles, HBV genotyping may provide additional prognostic information, particularly regarding immune escape mutations and long-term hepatocellular carcinoma risk. Routine hepatitis B screening prior to invasive procedures remains essential, as it may identify previously unrecognized chronic infection in asymptomatic individuals without identifiable high-risk exposures.

## Conclusion

This case illustrates a rare and clinically important serologic pattern of coexisting HBsAg and anti-HBs positivity in a patient with chronic hepatitis B infection**,** identified incidentally during routine screening and in the absence of prior vaccination or known risk factors. Anti-HBs positivity should not be assumed to indicate viral clearance or vaccination, and atypical serologic profiles require integration of serologic, virologic, and clinical data for accurate diagnosis and risk stratification. Routine hepatitis B screening prior to invasive procedures may uncover previously unrecognized chronic infection, allowing timely monitoring and preventive strategies. Clinicians should remain vigilant for such atypical presentations, as they carry important implications for long-term disease surveillance, therapeutic decision-making, and hepatocellular carcinoma risk. Specialist referral, comprehensive virologic assessment, and ongoing surveillance, including consideration of HBV genotyping, areessential to optimize patient management.

## Data Availability

The datasets used and/or analyzed during the current study are available from the corresponding author on reasonable request.

## Abbreviations

HBV: Hepatitis B virus
HCC: Hepatocellular carcinoma
HBsAg: Hepatitis surface antigen
HBsAb: Anti hepatitis B surface antigen antibodies
Anti-HBs: Anti hepatitis B surface antigen antibodies
CHB: Chronic hepatitis B
FIT: Fecal immunochemical testing
HBV DNA: HBV deoxyribonucleic acid
HCV Ab: Hepatitis C virus antibodies
HBc IgM: HBV core immunoglobulin M
HBc total Ab: HBV core total antibodies
R.R.: Reference range
HBV DNA PCR: HBV DNA polymerase chain reaction
AST: Aspartate aminotransferase
ALT: Alanine aminotransferase
LFTs: Liver function tests
U.S.: Ultrasound
CBD: Common bile duct
PV: Portal vein
IHBD: Intra hepatic biliary duct
CAP: Controlled attenuation parameter
IQR: Interquartile range
HBeAg: Hepatitis B e antigen

## References

[1] Polaris Observatory Collaborators. Global prevalence, cascade of care, and prophylaxis coverage of hepatitis B in 2022: a modelling study. Lancet Gastroenterol Hepatol. 2023;8(10):879–907. 10.1016/S2468-1253(23)00197-8. 37517414 10.1016/S2468-1253(23)00197-8

[2] Terrault NA, Lok AS, McMahon BJ, Chang KM, Hwang JP, Jonas MM, *et al*. Update on prevention, diagnosis, and treatment of chronic hepatitis B: AASLD 2018 hepatitis B guidance. Hepatology. 2018;67(4):1560–99. 10.1002/hep.29800. 29405329 10.1002/hep.29800 PMC5975958

[3] Jiang X, Chang L, Yan Y, Wang L. Paradoxical HBsAg and anti-HBs coexistence among chronic HBV infections: causes and consequences. Int J Biol Sci. 2021;17(4):1125–37. 10.7150/ijbss.55724. 33867835 10.7150/ijbs.55724 PMC8040313

[4] Ali MJ, Shah PA, Rehman KU, Kaur S, Holzmayer V, Cloherty GA, *et al*. Immune escape mutations are prevalent among patients with a coexistence of HBsAg and anti-HBs in a tertiary liver center in the United States. Viruses. 2024;16(5):713. 10.3390/v16050713. 38793596 10.3390/v16050713 PMC11125813

[5] Rezaee R, Poorebrahim M, Najafi S, Sadeghi S, Pourdast A, Alavian SM, *et al*. Impacts of the G145R mutation on the structure and immunogenic activity of the hepatitis B surface antigen: a computational analysis. Hepat Mon. 2016;16(2):e39097. 10.5812/hepatmon.39097. 27642350 10.5812/hepatmon.39097 PMC5018363

[6] Wang J, Ding W, Liu J, *et al*. Association of coexistent hepatitis B surface antigen and antibody with severe liver fibrosis and cirrhosis in treatment-naive patients with chronic hepatitis B. JAMA Netw Open. 2022;5(5):e2216485. 10.1001/jamanetworkopen.2022.16485. 35696167 10.1001/jamanetworkopen.2022.16485 PMC9194671

[7] Jin ZZ, Jin FF, Liu X, Liu N, Wen F, Lou JL. Coexistence of low levels of HBsAg and high levels of anti-HBs may increase risk of hepatocellular carcinoma in chronic hepatitis B patients with high HBV load. Braz J Infect Dis. 2019;23(5):343–51. 10.1016/j.bjid.2019.08.007. 31542378 10.1016/j.bjid.2019.08.007 PMC9427988

[8] Xu DZ, Yang HC, Liu XY, *et al*. Coexistence of hepatitis B surface antigen and antibody in patients with chronic hepatitis B virus infection: a systematic review and meta-analysis. J Viral Hepat. 2022;29(7):545–55. 10.1111/jvh.13668.

[9] Pu Z, Zheng X, Liu L, Lin D, Zhang H, Li J, *et al*. HBsAg and anti-HBs coexistence in patients with HBV in acute and chronic phases. Virus Res. 2025;355:199567. 10.1016/j.virusres.2025.199567. 40174727 10.1016/j.virusres.2025.199567 PMC12001098

[10] Pancher M, Désiré N, Ngo Y, Akhavan S, Pallier C, Poynard T, *et al*. Coexistence of circulating HBsAg and anti-HBs antibodies in chronic hepatitis B carriers is not a simple analytical artifact and does not influence HBsAg quantification. J Clin Virol. 2015;62:32–7. 10.1016/j.jcv.2014.11.015. 25542467 10.1016/j.jcv.2014.11.015

[11] Seo SI, Choi HS, Choi BY, Kim HS, Kim HY, Jang MK. Coexistence of Hepatitis B surface antigen and antibody to Hepatitis B surface may increase the risk of hepatocellular carcinoma in chronic Hepatitis B virus infection: a retrospective cohort study. J Med Virol. 2014;86:124–30. 10.1002/jmv.23779. 24127328 10.1002/jmv.23779

[12] Ayaz ÇM, Başpınar B, Güner R. Atipik Serolojik Profilli Hepatit B Hastalarının Klinik ve Laboratuvar Özellikleri. Clinical and laboratory characteristics of patients with hepatitis B with atypical serologic profiles. Viral Hepat J. 2022;28(3):94-s99. 10.4274/vhd.galenos.2022.2022-6-2.

[13] Lee WM, King WC, Schwarz KB, Rule J, Lok ASF. Prevalence and clinical features of patients with concurrent HBsAg and anti-HBs: evaluation of the Hepatitis B research network cohort. J Viral Hepat. 2020;27:922–31. 10.1111/jvh.13312. 32364641 10.1111/jvh.13312

[14] Fu X, Chen J, Chen H, Lin J, Xun Z, Li S, *et al*. Mutation in the S gene of Hepatitis B virus and anti-HBs subtype-non specificity contributed to the co-existence of HBsAg and anti-HBs in patients with chronic Hepatitis B virus infection. J Med Virol. 2017;89:1419–26. 10.1002/jmv.24782. 28198078 10.1002/jmv.24782

[15] Zhang JM, Xu Y, Wang XY, Yin YK, Wu XH, Weng XH, *et al*. Coexistence of Hepatitis B surface antigen (HBsAg) and heterologous subtype-specific antibodies to HBsAg among patients with chronic Hepatitis B virus infection. Clin Infect Dis. 2007;44:1161–9. 10.1086/513200. 17407033 10.1086/513200

